# Attenuation of intestinal inflammation in IL-10 deficient mice by a plasmid carrying *Lactococcus lactis* strain

**DOI:** 10.1186/s12896-020-00631-0

**Published:** 2020-07-23

**Authors:** Meritxell Zurita-Turk, Bianca Mendes Souza, Camila Prósperi de Castro, Vanessa Bastos Pereira, Vanessa Pecini da Cunha, Tatiane Melo Preisser, Ana Maria Caetano de Faria, Denise Carmona Cara Machado, Anderson Miyoshi

**Affiliations:** 1 Laboratório de Tecnologia Genética, Departamento de Biologia Geral, Instituto de Ciências Biológicas, Universidade Federal de Minas Gerai, Av. Antônio Carlos, 6627, Pampulha, 31, Belo Horizonte, MG 270-901 Brazil; 2 Laboratório de Imunobiologia, Departamento de Bioquímica e Imunologia, Instituto de Ciências Biológicas, Universidade Federal de Minas Gerai, Belo Horizonte, Minas Gerais Brazil; 3 Laboratório de Alergia e Inflamação, Departamento de Morfologia, Instituto de Ciências Biológicas, Universidade Federal de Minas Gerai, Belo Horizonte, Minas Gerais Brazil

**Keywords:** Inflammatory bowel diseases, Interleukin-10, *Lactococcus lactis*, pValac:*il-10*, IL-10-deficient mice

## Abstract

**Background:**

Inflammatory bowel diseases (IBD) are intestinal disorders characterized by inflammation in the gastrointestinal tract (GIT) and to date, no efficient treatments exist. Interleukin-10 (IL-10), one of the most important anti-inflammatory cytokines of the immune response, has been under study due to its potential for IBD therapy; however, systemic treatments lead to undesirable side effects and oral administration is limited due to its quick degradation. To avoid these bottlenecks, we previously engineered an invasive *Lactococcus lactis* (*L. lactis*) strain capable of delivering, directly to host cells, a eukaryotic DNA expression vector coding for IL-10 of *Mus musculus* (pValac:*il-10*) that diminished inflammation in two induced mouse models of intestinal inflammation. Thus, the aim of this study was to analyze its therapeutic effect in the IL-10-deficient mouse model (IL-10^−/−^) that spontaneously and gradually develops an inflammation that modifies the immune system and resembles Crohn’s disease (CD) in humans, and evaluate if it would also diminish and/or prevent the onset of this disease.

**Results:**

Oral administration of *L. lactis* MG1363 FnBPA+ (pValac:*il-10*) to IL-10^−/−^ mice not only led to IL-10 production by these, but consequently also diminished the severe development of the disease, with animals showing lower macroscopic scores and histological damages, increased IL-10 levels and tendency to lower pro-inflammatory cytokine levels.

**Conclusions:**

The results of this study, together with the previously published ones using this DNA delivery-based strategy, show that it is capable of creating and maintaining an anti-inflammatory environment in the GIT and thus effectively diminish the onset of inflammation in various mouse models.

## Background

Inflammatory bowel diseases (IBD), including Crohn’s disease (CD) and ulcerative colitis (UC), are intestinal disorders characterized by chronic inflammation in the gastrointestinal tract (GIT), and although sharing clinical and pathological features, they differ in their histological aspect and cytokine profiles [1]. The exact etiology and pathogenesis of these diseases are unclear despite much research in the last decades, but it is nowadays generally accepted that they are caused by deregulation of the mucosal immune system towards the native intestinal microbiota in genetically susceptible individuals, resulting in an inappropriate and excessive activation of the intestinal immune system [2]. As no unique and defined causal agent is responsible for the development of these diseases, current treatment proposals, including anti-inflammatory drugs, immunosuppressants and antibiotics, only improve the patient’s quality of life while presenting serious side effects and no cure, revealing that better, cheaper and longer lasting treatments are necessary [3].

IL-10-deficient mice (IL-10^−/−^) not raised in a defined specific-pathogen-free (SPF) environment develop a spontaneous gut inflammation, called enterocolitis, that is characterized by weight loss and anemia at the age of 4–6 weeks, lethal up to the age of 3 months [4], and is most severe in the colon, involving the small intestine to a lesser extent. The typical inflammatory lesions of this disease are discontinuous and transmural, and include epithelial hyperplasia, crypt abscesses, ulcers, mucin depletion and bowel wall thickening with infiltration of lymphocytes, plasma cells, macrophages, eosinophils and neutrophils [4]; its development is mediated by CD4+ T cells and an uncontrolled Th1 response [5].

Interleukin-10 (IL-10) is one of the most important anti-inflammatory cytokines in shaping mucosal immune responses in the gut [4, 6]. This cytokine is produced by regulatory T cells, epithelial cells, macrophages, dendritic cells and B1 cells [7] and therefore presents many important properties, including stimulation of B-cell differentiation and immunoglobulin secretion [8] and suppression of macrophage activation to inhibit inflammatory cytokines production. IL-10 is a good therapeutic candidate against IBD due to its immunosuppressive activity and central role in downregulating inflammatory cascades [9, 10]; systemic and oral treatments with recombinant human IL-10 were first tested in CD patients but presented important drawbacks, such as short half-life and extreme sensitivity of the gastrointestinal tract [11–14]. Steidler et al. developed an IL-10-producing *Lactococcus lactis* (*L. lactis*) strain [15] and although initial studies showed promising results, no statistically significant difference in mucosal healing between patients receiving the recombinant strains and a placebo were observed [16].

To overcome IL-10’s sensitivity and survival in the gastrointestinal tract, our research group used a novel strategy based on the use of the eukaryotic DNA expression vector pValac, constructed by ourselves in 2009, and a *L. lactis* strain to deliver and trigger DNA expression by epithelial cells of the host. Basically, after oral administration of the bacterial strain carrying the plasmid of interest, these enter target cells, bacteria suffer lysis and plasmids are liberated to the cytoplasm and transferred into the cell’s nucleus. Subsequently, the cell’s machinery is responsible for the expression of the ORF of interest, translation and protein synthesis [17]. Advantages of this strategy include the targeting of mucosal immunity, simple technology and low cost as well as safe use of *L. lactis*, as this strain does not colonize the gastrointestinal tract and transits it between 2 and 3 days, not becoming part of the normal gut flora.

The constructed *L. lactis* strain carrying the therapeutic pValac:*il-10* plasmid delivers it directly to the host’s cells. This strategy does not only ensure a more effective and direct delivery of the therapeutic plasmid but consequently a higher and more efficient IL-10 in situ production. This strategy successfully showed its anti-inflammatory effect and intestinal inflammation prevention in two induced inflammatory mouse models: TNBS (2,4,6-Trinitrobenzenesulfonic acid) and DSS (Dextran Sodium Sulphate) [18, 19], showing to be innovative and promising for the therapeutic IBD treatment.

In this line, the aim of the present work was to evaluate the *L. lactis* MG1363 FnBPA+ (pValac:*il-10*) strain as therapy in IL-10^−/−^ mice and evaluate its capacity to diminish and/or prevent the onset of enterocolitis.

## Results

### Evaluation of the animals’ weight

**Fig. 1:**
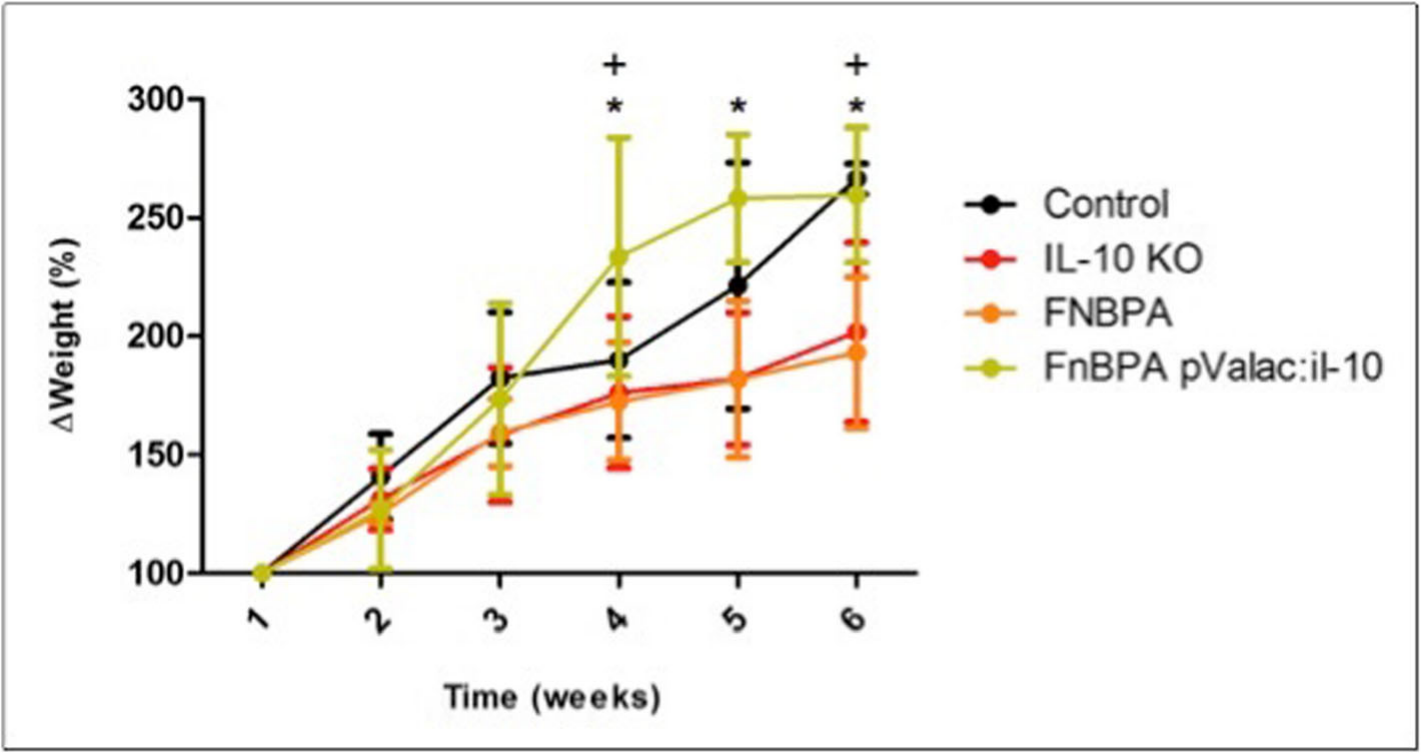
Corporal weight variation (%) of mice along the 6 weeks of experimentation. Healthy control group: negative control of intestinal inflammation, KO: IL-10^−/−^ mice - positive control of intestinal inflammation; FnBPA: IL-10^−/−^ mice that received the *L. lactis* MG1363 FnBPA+ strain; pValac:*il-10*: IL-10^−/−^ mice that received the *L. lactis* MG1363 (pValac:*il-10*) strain. +: Experimental group (control) whose percentage of corporal weight is statistically different from those of group pValac:*il-10* in week 4 and groups KO and FnBPA in week 6. *: Experimental group (pValac:*il-10*) whose corporal weight percentage is statistically different from those of groups KO and FnBPA in weeks 4, 5 and 6 (*p* < 0.001). The data is presented as the mean ± SD

The animals’ weight was assessed throughout the 6 weeks of experimentation, starting at the age of 2 weeks. All experimental groups presented an initial similar weight gain, according to the normal growth of these animals and development of the disease (Fig. 1). However, IL-10^−/−^ mice from the KO and FnBPA groups showed an overall lower weight gain after 2 weeks of experimentation, when compared with the healthy control and the pValac:*il-10* groups. On the other hand, IL-10^−/−^ mice from the pValac:*il-10* group showed rapid weight gain which equaled that of the healthy control group after the 6 weeks of experimentation. Moreover, this group also showed statistically significant differences with the KO and FnBPA groups in weeks 4, 5 and 6 (*p* < 0.001).

### Macroscopic and histologic evaluation

**Fig. 2:**
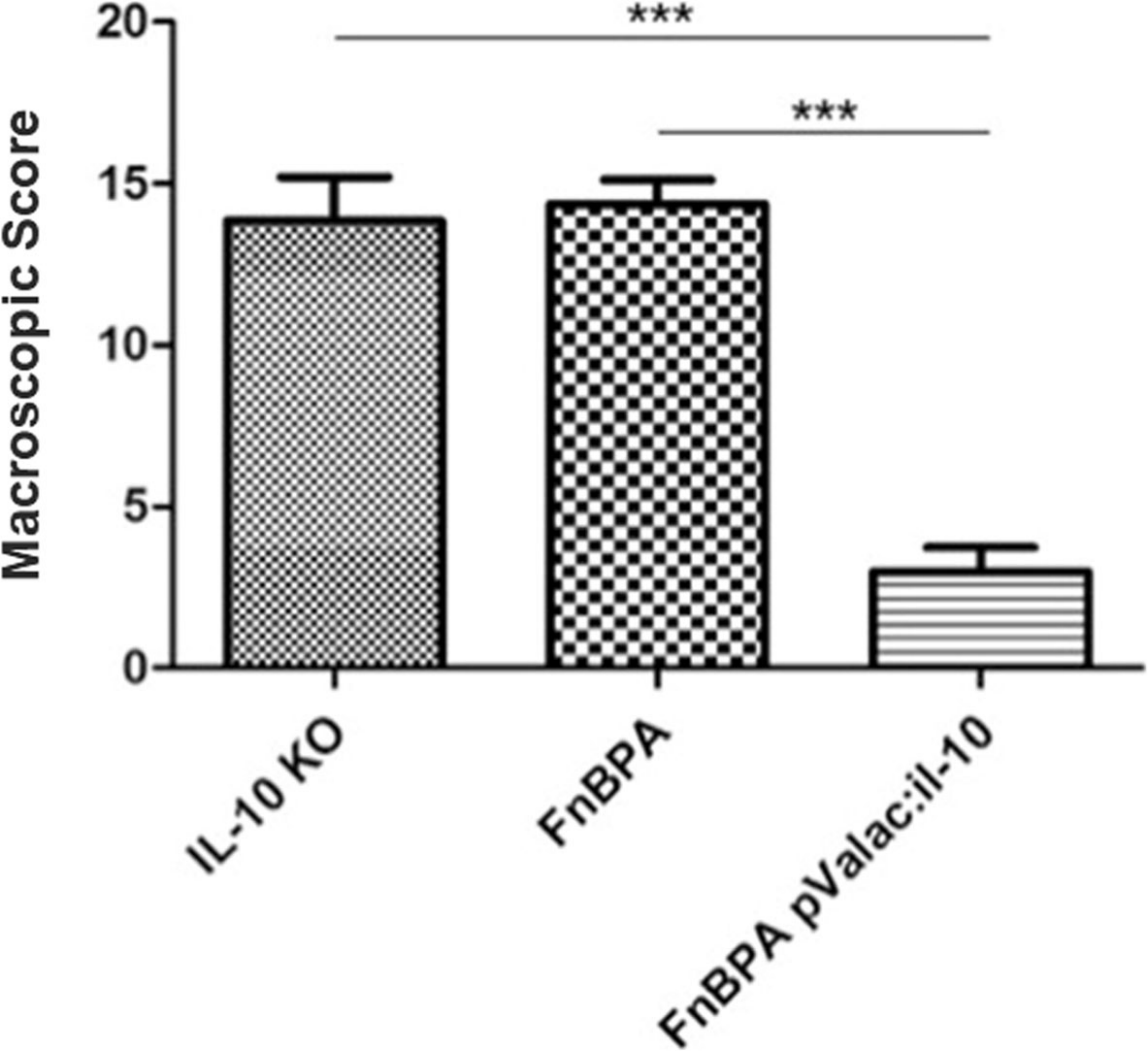
Macroscopic score of the different groups. Bars represent the mean *N* = 9 ± SD. Asterisks represent statistical significance (***p < 0.0001)

**Fig. 3:**
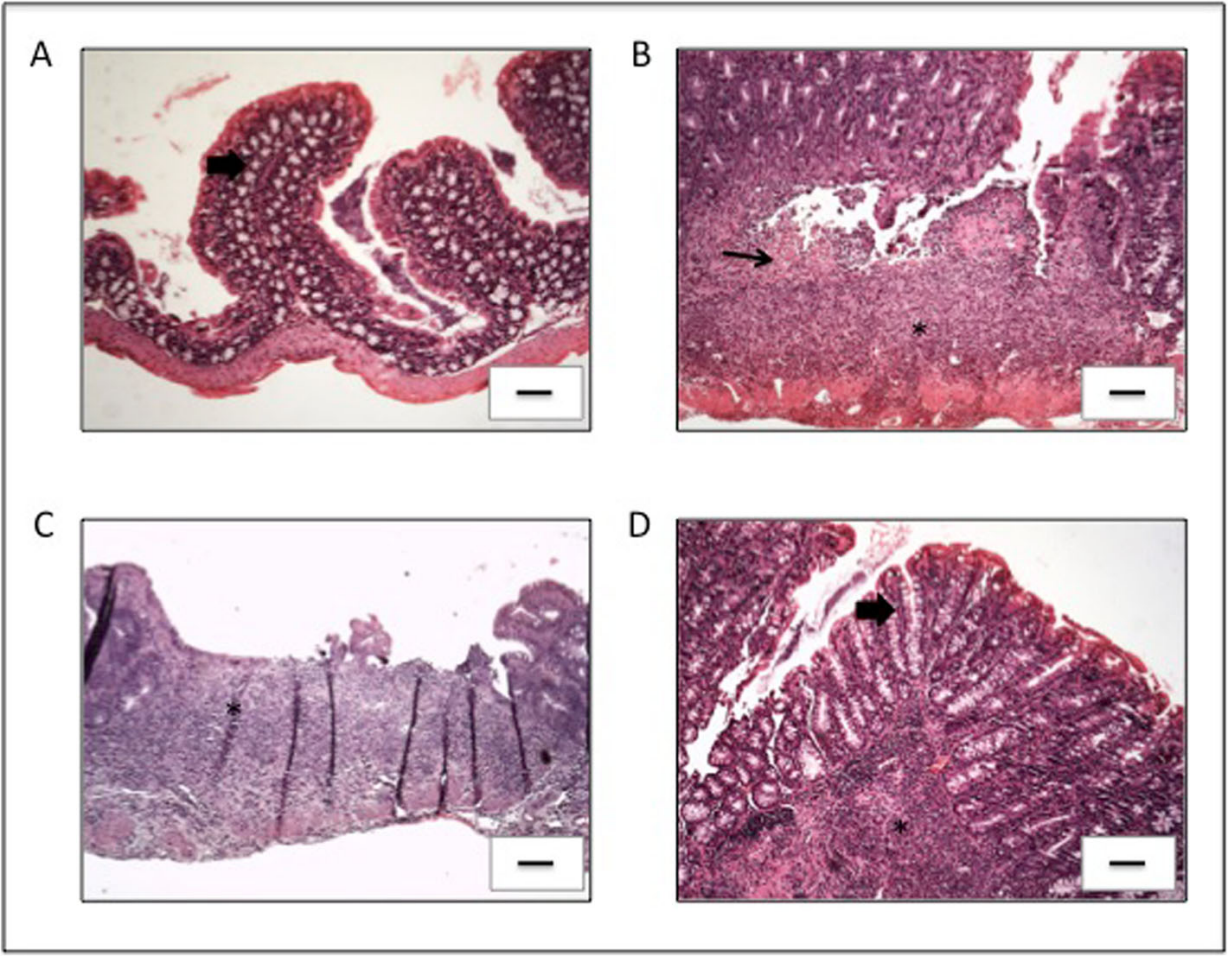
Histopathology of changes in the AC of all experimental groups. **a** Control group; **b** KO group; **c** FnBPA group and **d** pVala:*il-10* group. Presence of inflammatory infiltrate (asterisks) and mucosal lesion (black thin arrow) can be observed in varied degrees of intensity. The thick arrow highlights the preserved mucosal architecture with the presence of goblet cells. The strains were obtained from the tissue of AC stained with HE. The bar in each image represents 100 μm

The healthy control group presented neither macroscopic nor histologic intestinal lesions (Figs. 2 and 3a) and is not shown in Fig. 2 as its macroscopic score was 0. Hematoxylin-eosin (HE) staining of samples of the ascending colon (AC) showed that this group presented a normal histological architecture with an intact mucosa, thin submucosal and serosal layers, muscular layer thickness compatible with the analyzed segment and normal proportion of goblet cells. No signs of degenerative, vascular, inflammatory or pathological cell proliferation processes were observed (Fig. 3a).

All IL-10^−/−^ mice, except those from the pValac:*il-10* group, presented similar macroscopic and histologic damage scores regarding the samples of their AC (Figs. 2 and 3b-c). It was possible to observe a compromised histological architecture with moderate inflammatory infiltrate in the mucosa and presence of mononuclear cells. Areas of erosion and reduction of goblet cells were also observed, as well as moderate inflammatory infiltrate in the submucosal layer and oedema. No evident alterations were observed in the muscular and the serosa layer (Fig. 3b and c). These results show that the disease strongly affected the AC region.

The pValac:*il-10* group, however, showed that administration of *L. lactis* MG1363 FnBPA+ (pValac:*il-10*) to IL-10^−/−^ mice was capable of strongly attenuating the pathology’s development, presenting a much lower macroscopic damage score and statistically significant differences with the KO and FnBPA groups (Fig. 2). Furthermore, the pValac:*il-10* group also showed significant improvement of histological patterns: the mucosa of the AC of this group presented light inflammation in less than 40% of animals, with no inflammatory alteration in the rest, no present signals of erosion nor depletion of goblet cells and no inflammatory infiltrate in the submucosal layer (Fig. 3d).

**Fig. 4:**
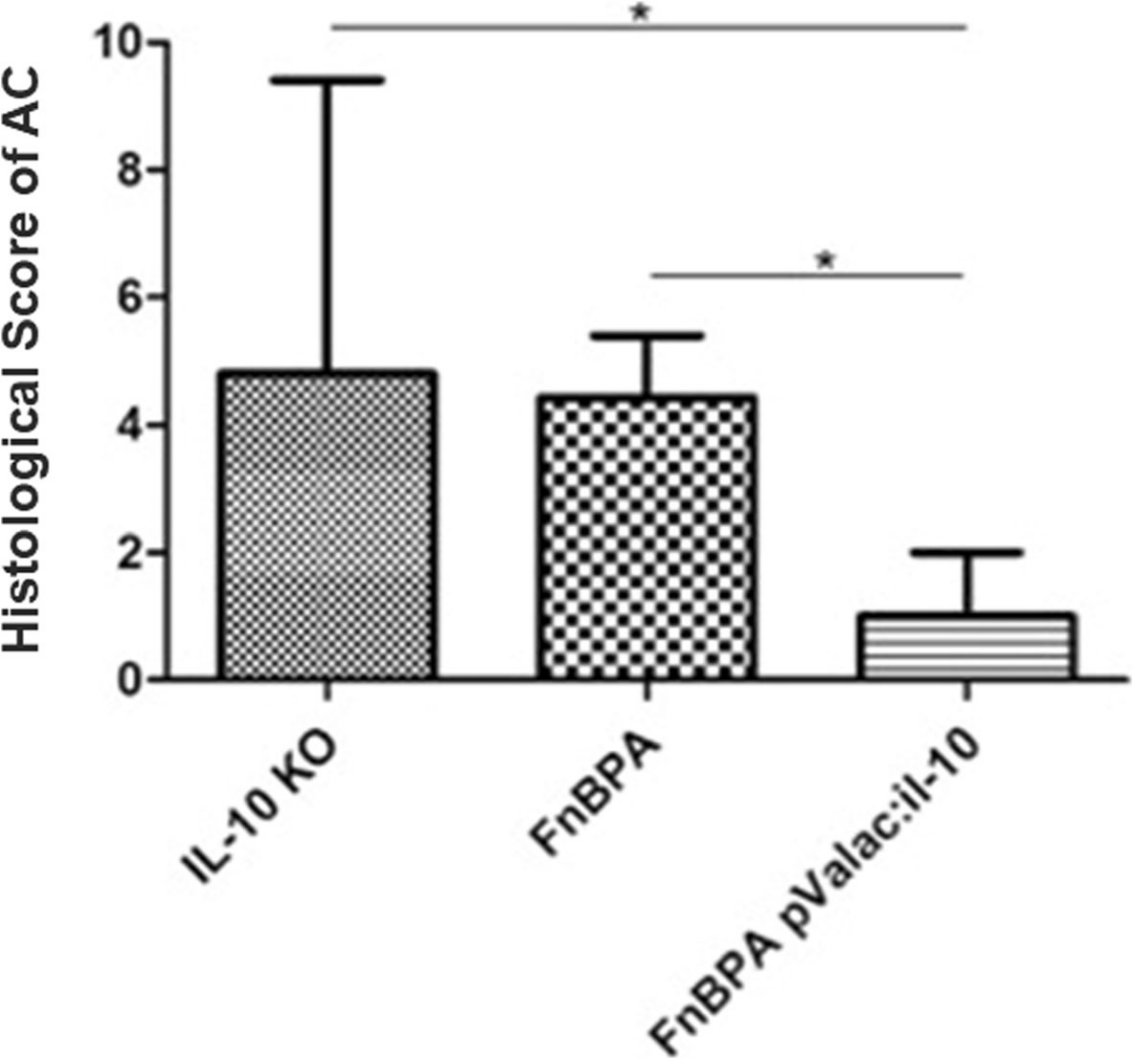
Histological score of AC samples of all groups. Bars represent the mean *N* = 9 ± SD. Asterisks represent statistical significance (*p < 0.05)

After evaluation of the histopathological damage of AC samples, the histological score was performed, and as expected, the IL-10^−/−^ control and *L. lactis* MG1363 FnBPA+ groups showed statistically significant differences with the *L. lactis* MG1363 FnBPA+ (pValac:*il-10*) group (Fig. 4).

**Fig. 5:**
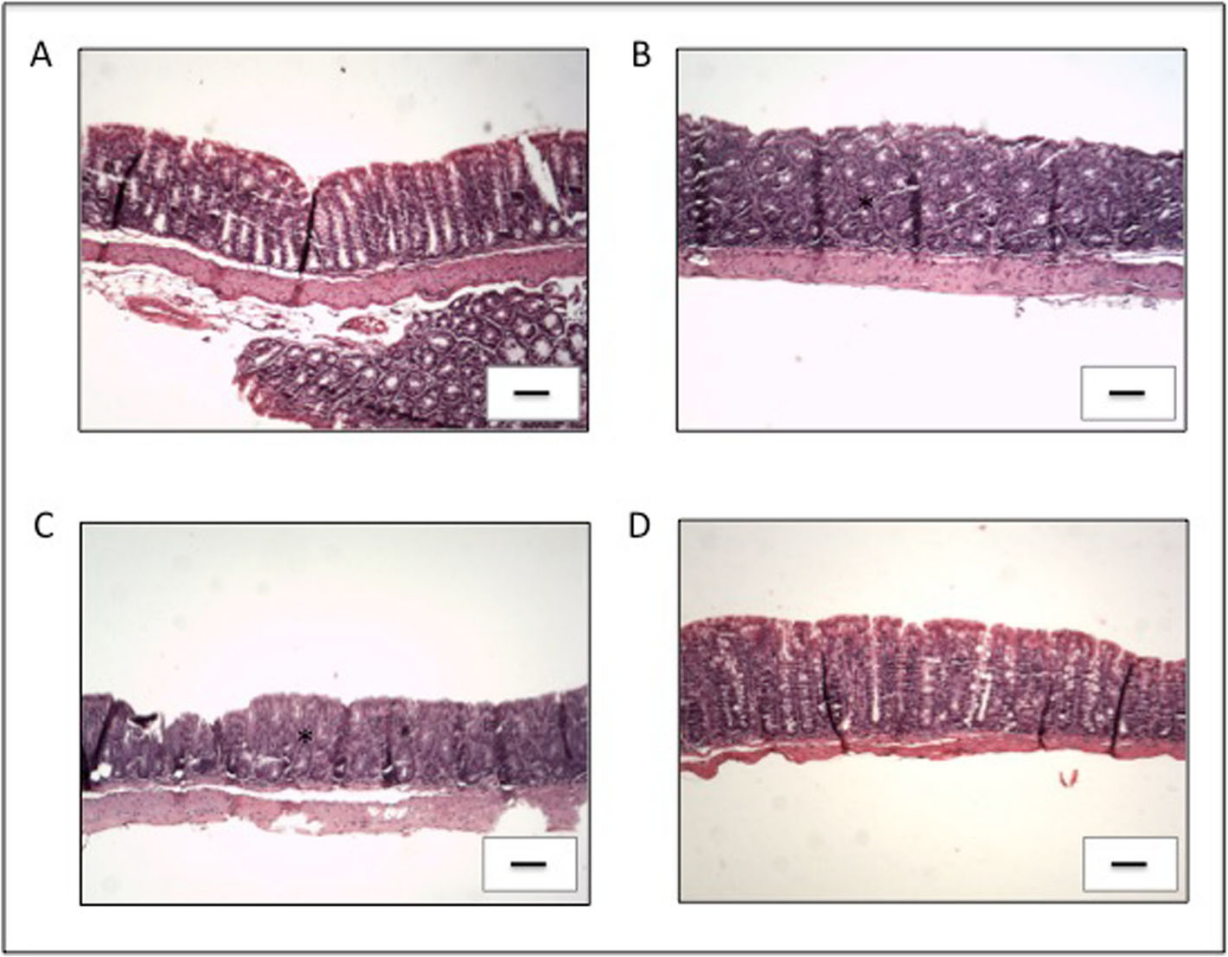
Histopathology of changes in the DC of all experimental groups. **a** Control group; **b** KO group; **c** FnBPA group and **d** pVala:*il-10* group. Presence of inflammatory infiltrate (asterisks) can be observed in varied degrees of intensity. The strains were obtained from the tissue of DC stained with HE. The bar in each image represents 100 μm

HE staining of samples of the descending colon (DC) of the healthy control group showed that all fragments of this group presented patterns of normality, with normal histological architecture and integral mucosa without erosion and with presence of goblet cells in the adequate proportion. The muscular layer thickness observed, thin submucosal and serosal layers, were compatible with the analyzed segment and no signs of degenerative or pathological cell proliferation processes were observed. Some few animals presented a light inflammatory infiltrate in the region of the mucosa and oedema in the submucosa (Fig. 5a).

IL-10^−/−^ animals of the KO and FnBPA groups presented a compromised histological architecture. HE staining of DC samples of these groups showed moderate inflammatory infiltrate in the mucosa, marked by mononuclear cells; however, no areas of erosion, ulceration or with goblet cells reduction were observed. Moderate oedema and light infiltrate were observed in the submucosa region, with no evident alteration in the muscular and serosa layers (Fig. 5b and c). These results show that the disease also strongly affects the descendant region of the colon.

IL-10^−/−^ animals that received the *L. lactis* MG1363 FnBPA+ (pValac:*il-10*) strain showed an attenuated pathologic pattern, with light inflammatory infiltrate in the mucosa and no observed alteration in the epithelium. Moreover, alterations in the submucosa (oedema and infiltrate) were considered light or even absent in some animals (Fig. 5d).

**Fig. 6:**
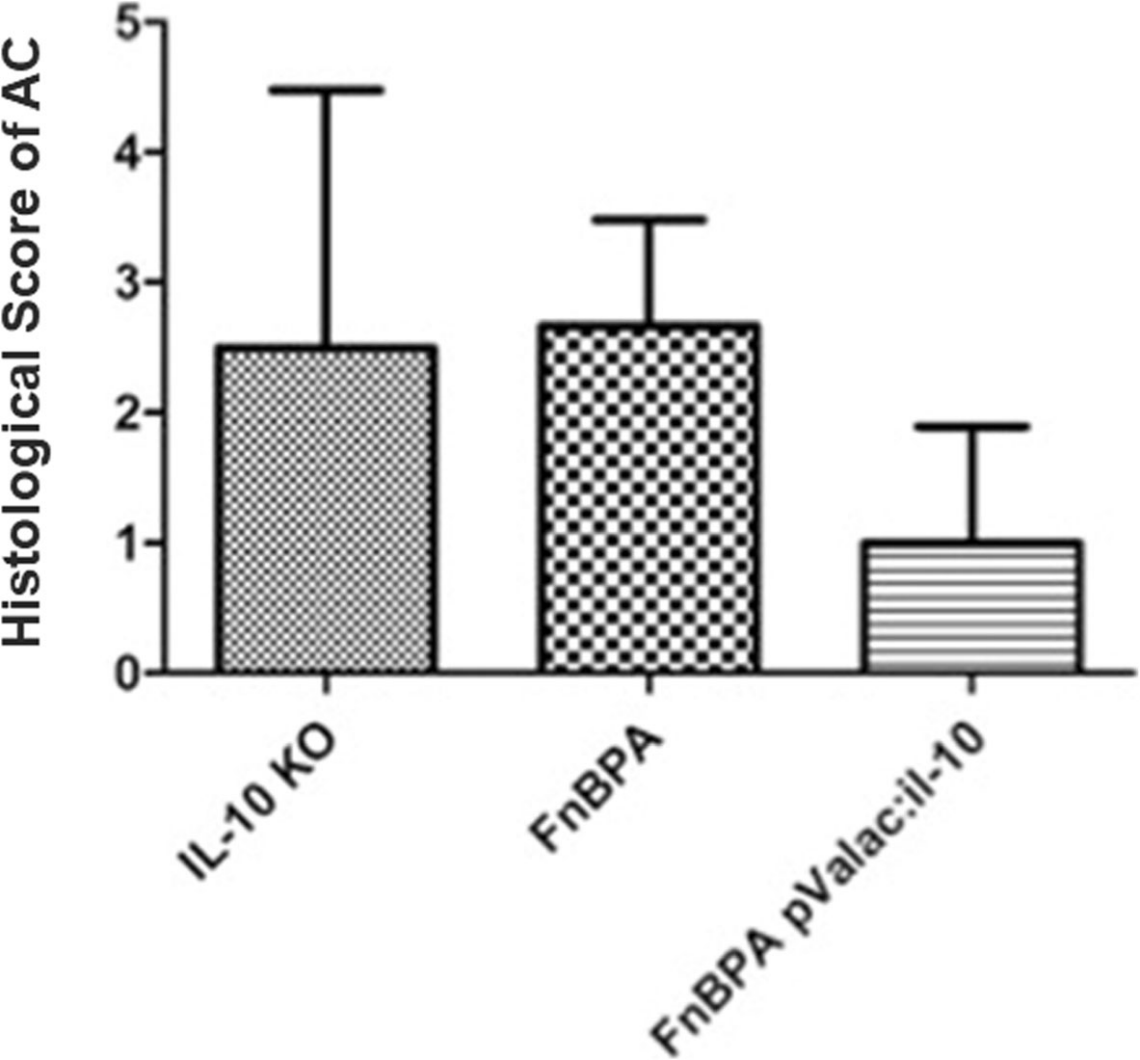
Histological score of DC samples of all groups. Bars represent the mean *N* = 9 ± SD

Again, after analysis of histopathological damages of DC samples, the histological score of all groups was realized and showed, as expected, that the KO and FnBPA groups showed major histological damage scores than the *L. lactis* MG1363 FnBPA+ (pValac:*il-10*) group (Fig. 6).

### Administration of *L. lactis* MG1363 FnBPA+ (pValac:*il-10*) results in IL-10 production in all evaluated tissues

**Fig. 7:**
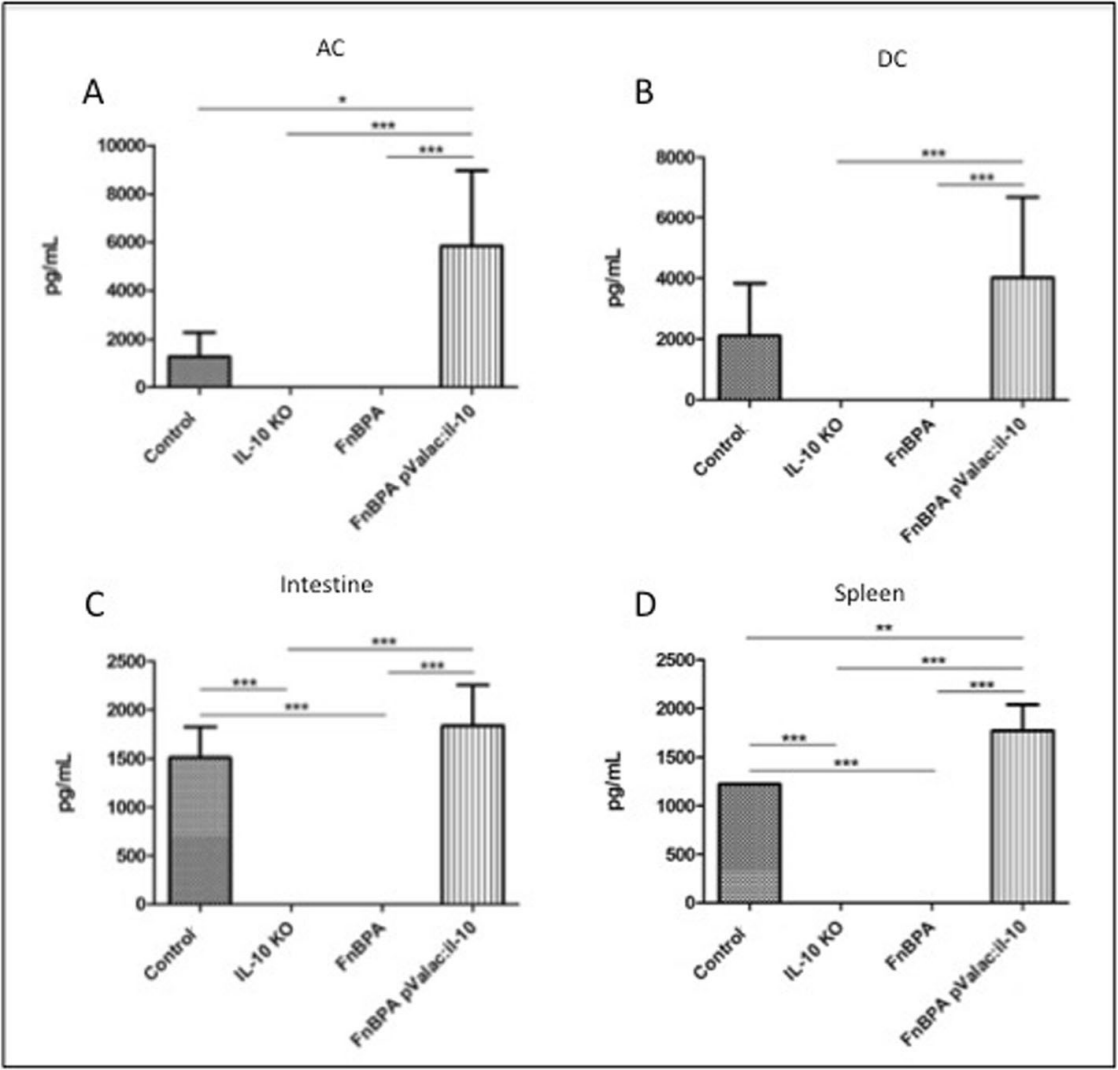
Administration of the pValac:*il-10* plasmid resulted in IL-10 production by IL-10^−/−^ mice in all evaluated tissues. Levels of IL-10 in (**a**) AC, (**b**) DC, (**c**) small intestine and (**d**) spleen of control, KO, FnBPA and pValac:*il-10* groups. Bars represent the mean *N* = 9 ± SD. The asterisks represent statistical significance (*p < 0.05, **p < 0.001 or ***p < 0.0006)

All IL-10^−/−^ mice from the groups that did not receive any treatment (KO and FnBPA) did not produce IL-10 (Fig. 7). This in turn resulted in statistically significant differences (*p* < 0.0006) with the control group in the small intestine and spleen (Fig. 7c-d) and with the pValac:*il-10* group in all evaluated tissues. As expected, *L. lactis* MG1363 FnBPA+ (pValac:*il-10*) administration to IL-10^−/−^ mice led to IL-10 production in all evaluated tissues. It was quite surprising to observe that IL-10 levels in the control group were lower than in the pValac:*il-10* group, especially in the AC and spleen (Fig. 7a and d), where these were even statistically significant (*p* < 0.05 and *p* < 0.001, respectively).

### Administration of the pValac:*il-10* plasmid tends to alter the production of IL-6 and IFN-γ cytokines

**Fig. 8:**
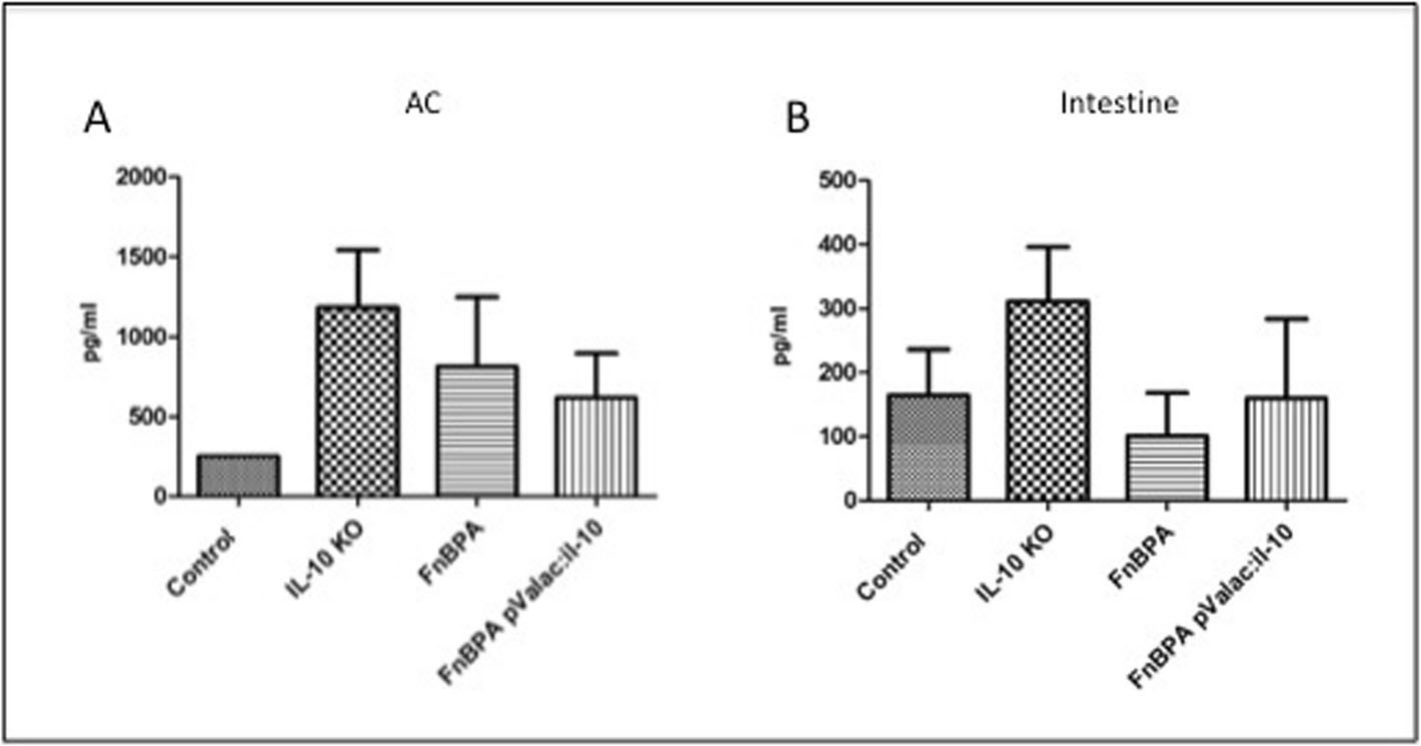
Administration of the pValac:*il-10* plasmid modulates the production of IL-6 in all tissues evaluated. Levels of IL-6 in (**a**) AC and (**b**) small intestine of control, KO, FnBPA and pValac:*il-10* groups. Bars represent the mean *N* = 9 ± SD

*L. lactis* MG1363 FnBPA+ (pValac:*il-10*) administration to IL-10^−/−^ mice resulted in lower IL-6 production levels in the AC when compared to both the KO and FnBPA groups; however, these were not statistically significant (Fig. 8a). In the small intestine, although not being statistically significant, IL-6 levels of the pValac:*il-10* were lower than in the KO group but slightly higher than in the FnBPA group (Fig. 8b).

**Fig. 9:**
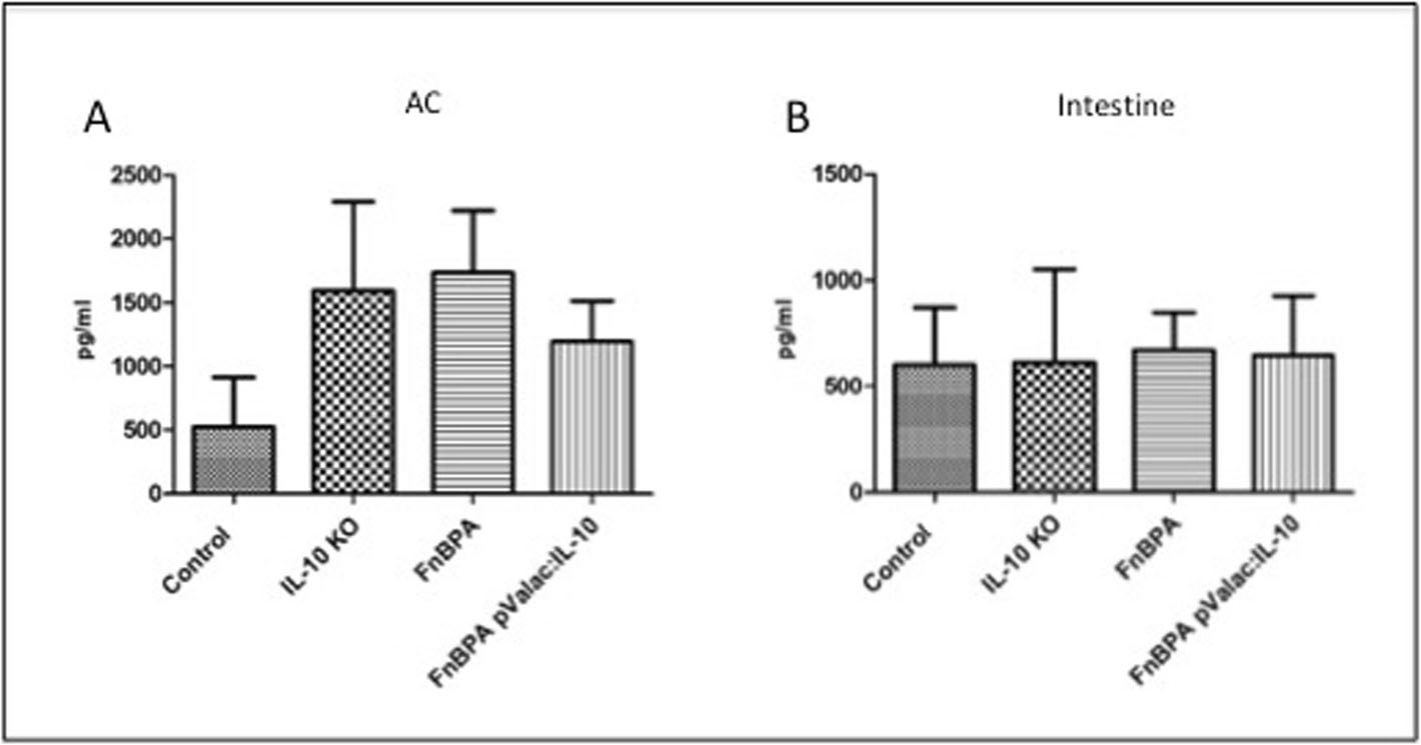
Effect of the pValac:*il-10* plasmid administration in IFN-γ production. Levels of IFN-γ in (**a**) AC and (**b**) small intestine of control, KO, FnBPA and pValac:*il-10* groups. Bars represent the mean *N* = 9 ± SD

In relation to IFN-γ production, the pValac:*il-10* group presented lower levels both in the AC and small intestine with no statistically significant difference with the other groups (Fig. 9). Again, regarding lower IL-6 levels in the pValac:*il-10* group, administration of the *L. lactis* MG1363 FnBPA+ (pValac:*il-10*) strain to IL-10^−/−^ mice tended to reduce IFN-γ production levels.

### No bacterial translocation was observed after oral administration

No bacterial translocation was detected in the spleen and liver up to 24 h following oral administration of bacterial strains (Table 1).

**Table 1:** Bacterial translocation

	Time after gavage				
Bacterial Translocation	2 h	4 h	6 h	12 h	24 h
Liver	0	0	0	0	0
Spleen	0	0	0	0	0

## Discussion

Much research in the last decades has focused in unveiling the etiology and pathogenesis of IBDs to develop therapies for their treatment, as no cure for them exists. Many strategies have based their research on the use of IL-10 due to its anti-inflammatory properties and key role in the development of the disease, as evidenced by IL-10^−/−^ mice, which develop enterocolitis, a severe disease in the colon characterized by weight loss, anemia and strong inflammatory lesions, apparent already at 4 weeks of age and lethal up to the age of 3 months, when maintained under conventional conditions [4].

The research and development of therapies using IL-10 are based on its delivery directly at the intestinal mucosa, as its correct administration and targeting to the sites of inflammation has been the major bottleneck for successful results. These strategies have included the use of polymer-based microparticles [20] and the use of bacterial strains capable of secreting human IL-10 [21–23].

As a new and innovative approach for IL-10 production at inflammation sites, our research group developed a new strategy that involved the use of a *L. lactis* strain expressing FnBPA [17, 24], that has the capacity to efficiently internalize and trigger recombinant DNA expression by human epithelial cells, as delivery vehicle of a eukaryotic expression vector coding for IL-10, pValac:*il-10*, directly to the host’s cells in the GIT for recombinant IL-10 in situ production. This strategy does not only ensure a more effective and direct delivery of the therapeutic plasmid but consequently a higher and more efficient IL-10 production. This strategy showed to be capable of preventing inflammation in the TNBS-and DSS-induced mouse models [18, 19].

In this research, 2 weeks old mice received the pValac:*il-10* plasmid during six consecutive weeks to evaluate its therapeutic effect. Macroscopic and histological analysis showed that non-treated mice presented an overall lower weight gain during this period while those under treatment showed a rapid weight gain equaling that of the healthy control group. This growth retardation in IL-10^−/−^ mice could be explained by the severe lesions in the GIT that led to disturbed nutrient absorption, contrary to the animals that received the therapy and consequently produced IL-10. Regarding the inflammatory damage in the AC and DC, mice from the KO and FnBPA groups presented higher macroscopic damage scores and important lesions in the colon while IL-10^−/−^ mice that received the pValac:*il-10* plasmid showed an attenuated pathology’s development and as such a much lower macroscopic damage scores, showing that IL-10 administration does prevent a worse onset of inflammation and therefore disease development.

Other important parameters measured during this period were the levels of pro-inflammatory and anti-inflammatory cytokines. The normal gastrointestinal inflammatory response is tightly regulated, and the balance of pro-inflammatory and anti-inflammatory cytokines produced by CD4+ Th1 and Th2 cells is an important part of this regulatory process [25]. Intestinal inflammation in IL-10^−/−^ mice is characterized by dysregulation of the immune system with regulatory T cells incapable of developing or functionally impaired in the absence of IL-10, leading to activation of Th1 cells and overproduction of pro-inflammatory cytokines resulting in chronic inflammation [5]. However, administration of the pValac:*il-10* plasmid led to IL-10 production by mice naturally incapable of producing this cytokine, which, as a consequence, lowered the disease development when compared to IL-10^−/−^ mice that did not receive any treatment. As already mentioned, IL-10 is essential to control intestinal immune responses. Moreover, some evidence suggests that IL-10 can inhibit the translocation of nuclear factor-kB, inhibiting the immediate-early pro-inflammatory response [26] and downregulate acute inflammatory responses.

This IL-10 production altered the production of pro-inflammatory cytokines, such as IL-6, which presented lower levels in all tissues of treated mice. IL-6 is produced by T and B lymphocytes, which infiltrate inflammatory lesions in IL-10^−/−^ mice, and which presents high levels during IBDs, therefore playing a functional role in their pathogenesis [4]. Moreover, in IL-10^−/−^ mice, IL-6 can also be considered an inflammatory mediator responsible for maintaining/increasing the intestinal inflammation [5] and its levels were therefore measured. On the other hand, IFN-γ, identified as a major mediator initiating colitis in IL-10^−/−^ neonates [5, 27], showed increased levels in IL-10^−/−^ mice as a result of failed regulatory T cells production or even functionality in the absence of IL-10, suggesting that Th1 cells are activated very early in the disease process [27]. A higher IFN-γ production is related to a more severe colitis [28].

To confirm that the genetically modified *L. lactis* strain is indeed transient through the gastrointestinal tract and does not colonize it, bacterial translocation to the spleen and liver was evaluated. No bacterial translocation was observed in these organs at any of the evaluated times (2 h, 4 h, 6 h, 12 h and 24 h) after oral administration. This is a particularly important safety parameter regarding human use.

The work here presented represents the last step in validating this DNA delivery strategy using the *L. lactis* MG1363 bacterial strain. When first constructing the pValac:*il-10* plasmid, this was tested for its transfection efficiency and subsequent IL-10 production and secretion by eukaryotic cells through ELISA, confocal microscopy and FACS [18]. Following, this *L. lactis* MG1363 FnBPA+ (pValac:*il-10*) strain was tested in two animal models to evaluate not only the success of the strategy in delivering the pValac*il-10* plasmid and following in situ IL-10 production and secretion by the animals cells, but also its immunomodulatory potential in diminishing the onset and development of intestinal inflammation due to increased IL-10 production, when compared with the control groups*.* In 2013, del Carmen and co-workers demonstrated that the severity of TNBS-induced intestinal inflammation decreased in mice after administration of this strain as a result of an increase in IL-10 levels and decrease in IFN-γ and IL-17 levels, when compared to the not treated groups [18]. Later, Zurita-Turk et al. demonstrated that administration of this *L. lactis* strain resulted in decreased severity of DSS-induced intestinal inflammation, probably also due to increased IL-10 levels and decreased IL-6 levels [19]. These studies showed that direct IL-10 production at inflammation sites produced an anti-inflammatory environment, as well as downregulated Th1-and Th17-mediated inflammation, capable of diminishing the onset and severity of the disease.

Thus, the main interest when testing this bacterial strain in the IL-10^−/−^ model was to evaluate if animals would produce IL-10 and consequently diminish the development of the intestinal inflammation and as such, validate the efficiency of this DNA delivery strategy. The results here presented are very exciting, as they demonstrate that our strategy leads to IL-10 production by mice that are naturally not able to, as well as higher production when compared to healthy mice capable of normally producing this cytokine, however, at a lower rate.

However, this current study presents one research limitation: FACS analyses were not performed. FACS analyses would have brought better knowledge regarding the mechanism of pValac:*il-10* delivery and uptake/invasion into eukaryotic cells, as well as which are the IL-10-producing cell populations. From the obtained results it can be stated that the proposed strategy was successful, as the pValac:*il-10* plasmid was delivered to eukaryotic cells in IL-10^−/−^ mice and subsequent IL-10 production was responsible diminish the development of intestinal inflammation. Nevertheless, the exact mechanism and which cells populations were involved, were not elucidated. In this context, future studies are required to answer these current research limitations.

## Conclusions

To evaluate the therapeutic capacity in preventing the onset of spontaneous enterocolitis in IL-10^−/−^ mice, *L. lactis* MG1363 FnBPA+ (pValac:*il-10*) was administered to 2-week old IL-10^−/−^ mice for six consecutive weeks. One feature characterizing the development of this disease is low weight gain, and mice receiving our therapy showed normal weight gain along the 6 weeks of treatment, comparable to the healthy control group. Moreover, these animals also showed overall lower macroscopic and histological damages, both in the AC and DC, when compared to not treated IL-10^−/−^ mice, showing capability in attenuating the pathology’s development and improving histological patterns. Moreover, tendency to lower IL-6 and IFN- γ levels were observed in the AC and small intestine, probably due to IL-10 production.

All these results show that administration of the *L. lactis* MG1363 FnBPA+ strain carrying the therapeutic pValac:*il-10* plasmid attenuated the development of spontaneous intestinal inflammation in IL-10^−/−^ mice by maintaining an anti-inflammatory environment in the GIT. Thus, administration of this strain led to IL-10 production by these animals and in this way diminished the onset of inflammation. These results, together with those presented in the TNBS- and DSS-induced inflammatory models, strengthen the potential of this strategy in the therapeutic treatment of IBD.

## Methods

### Bacterial strains, growth conditions and plasmid

The bacterial strains and plasmid used in this work are listed in Table 2. *L. lactis* strains were grown in M17 medium (Difco, Sparks, MD, USA) supplemented with 0.5% glucose (GM17) at 30 °C without shaking and bacterial selection was performed by addition of 10 μg/mL of chloramphenicol (Cm) and/or 5 μg/mL of erythromycin (Ery). *L. lactis* cultures were grown until an OD_600_ of 1.0–1.2 and stocked in glycerol 80% (1:4); on animal feedings day, doses were centrifuged to eliminate any traces of antibiotic and medium and resuspended in 1 mL of saline solution (0.15 M NaCl).

**Table 2:** Bacterial strains and plasmid

**Bacterial strain**	**Characteristics**	**Font**
*Lactococcus lactis* MG1363 FnBPA+	*L. lactis* MG1363 strain expressing FnBPA of *S. aureus*	[29]
*Lactococcus lactis* MG1363 FnBPA+ (pValac:*il-10*)	*L. lactis* MG1363 strain expressing FnBPA of *S. aureus* carrying the pValac:*il-10* plasmid	[18]
**Plasmid**	**Characteristics**	
pValac:*il-10*	(pCMV/CmR/RepA/RepC/IL-10)	[18]

### Animals

To evaluate the therapeutic capacity of the *L. lactis* MG1363 FnBPA+ (pValac:*il-10*) strain in diminishing and/or preventing the onset of enterocolitis, wild-type and IL-10^−/−^ mice on a 129Sv/Ev genetic background were used, as these are the most susceptible to colitis development with homogeneous clinical manifestation [5]. As these animals develop a spontaneous and gradual intestinal inflammation when not maintained in SPF conditions, mice were, for experimental procedures, maintained in collective cages in conventional conditions in an environmentally controlled room with a 12-h light/dark cycle and free access to water and food.

For experimental procedures, experimental groups were adjusted in relation to the animal’s weight at the age of 2 weeks and consisted of: i) healthy control group, ii) IL-10^−/−^ control group (KO group) and the IL-10^−/−^ groups that received the iii) *L. lactis* MG1363 FnBPA+ strain (FnBPA group) or the iv) *L. lactis* MG1363 FnBPA+ (pValac:*il-10*) strain (pValac:*il-10* group). As the disease is already apparent at the age of 4 weeks, the in vivo assays started with 2-week old mice that received intragastrically, over six consecutive weeks, 100 μL of the corresponding bacterial strain as suspension at a dose of 2 × 10^9^ CFU/100 μL or 100 μL of 0.9% saline solution (control groups) per day. After 6 weeks (at the age of 8 weeks), all mice were euthanized by overdose of a ketamine (100 mg/kg)/xylazine (10 mg/kg) mixture. The total n of this experimental procedure was 9.

Mice were kindly donated by Dr. Ana Maria Caetano de Faria from the Federal University of Minas Gerais (UFMG - Belo Horizonte, Brazil). All animal procedures and manipulations were approved by the Ethics and Research Committee on Animal Experiments (CEUA) of the Biological Institute of the Universidade Federal de Minas Gerais (UFMG - Belo Horizonte, Brazil) with protocol number 66/2011.

### Macroscopic and histologic assessment of enterocolitis

Animals’ weight was rigorously evaluated during the 6 weeks of experimental procedure to assess the macroscopic damage of the disease. After this time, as already described in the previous section, animals were euthanized by overdose of a ketamine/xylazine mixture and the colons were excised to assess colonic inflammation using a previously defined scoring system that evaluates the following features: presence or absence of adhesions, strictures and diarrhea (loose, watery stool), and bowel wall thickness (mm). As enterocolitis can affect the colon in different degrees, all colon samples were divided in ascendant and descendant regions for separate evaluation.

All colon samples were fixed in formalin and processed for histologic analysis. HE stained sections were blindly scored based on a semiquantitative scoring system previously described [30] that grades the following features: destruction extent of normal mucosal architecture (0: normal; 1: mild, 2: moderate and 3: extensive damage), presence and degree of cellular infiltration (0: normal; 1: mild, 2: moderate and 3: transmural infiltration), extent of muscle thickening (0: normal; 1: mild, 2: moderate and 3: extensive thickening), presence (1) or absence (0) of crypt abscesses and presence (1) or absence (0) of goblet cell depletion. Scores for each feature were summed up to a maximum possible score of 11.

### Tissue preparation and cytokine assay

Samples of AC and DC, small intestine and spleen were weighed and homogenized in phosphate-buffered saline (PBS) containing 0.05% (v/v) Tween-20, 0.1 mM phenylmethylsulphonyl fluoride, 0.1 mM benzethonium chloride, 10 mM EDTA and 20 KIU Aprotinin A using a tissue homogenizer (1 mL/0.1 g) for cytokine assays. Tissue fragments were then homogenized and centrifuged for 10 min at 600 g and 4 °C and supernatants were collected for cytokine assay, as previously described [31]. Briefly, 96-well plates (Nunc) were coated with purified monoclonal antibodies reactive with cytokines IL-6, IL-10 and IFN-*γ* (BD-Pharmingen) overnight at 4 °C. Wells were washed the next day, supernatants were added and plates incubated overnight at 4 °C. Following, biotinylated monoclonal antibodies against cytokines were added and plates were incubated for 2 h at room temperature. Color reactions were developed at room temperature with 100 μL/well of orthophenylenediamine (OPD) (1 mg/mL) (Sigma, St. Louis, MO, USA), 0.04% H_2_O_2_ substrate in sodium citrate buffer. Reactions were interrupted by addition of 20 μL/well of 2 N H_2_SO_4_ and absorbance was measured at 492 nm by an ELISA microplate reader (Bio-Rad). Results were expressed as concentration (pg/mL), according to the standard curve.

### Bacterial translocation

IL-10^−/−^ mice received 100 μL of *L. lactis* MG1363 FnBPA+ (pValac:*il-10*) intragastrically at a dose of 2 × 10^9^ CFU/100 μL. Spleen and liver were collected under strict aseptic conditions 2 h, 4 h, 6 h, 12 h and 24 h after bacterial administration. Tissues were macerated in 500 μL of 0.9% saline and 100 μL of this homogenate was plated in M17 medium (Sigma-Aldrich) supplemented with agar, 0.5% glucose, chloramphenicol (10 μg/mL) (Sigma-Aldrich) and erythromycin (5 μg/mL) (Sigma-Aldrich). Plates were incubated at 30 °C for 24 h.

### Statistical analysis

Statistical analyses were performed using the GraphPad Prism 5.0 software (San Diego, CA, USA). Student’s t-test or analysis of variance (ANOVA) followed by a Tukey comparison post-hoc test were used to assess the significance of differences among groups. Means were considered statistically different when *p* < 0.05 and all results were expressed as mean ± standard deviation (SD).

## Data Availability

The datasets used and/or analyzed during the current study are available from the corresponding author on reasonable request.

## Abbreviations

AC: Ascending colon
ANOVA: Analysis of variance
CD: Crohn’s disease
Cm: Chloramphenicol
DC: Descending colon
DSS: Dextran Sodium Sulphate
Ery: Erythromycin
GIT: Gastrointestinal tract
HE: Hematoxylin-eosin
IBD: Inflammatory bowel diseases
IL-10: Interleukin-10
IL-10^−/−^: IL-10-deficient mouse model
*L. lactis*: *Lactococcus lactis*
OPD: Orthophenylenediamine
PBS: Phosphate-buffered saline
SD: Standard deviation
SPF: Specific-pathogen-free
TNBS: 2,4,6-Trinitrobenzenesulfonic acid
UC: Ulcerative colitis
UFMG: Universidade Federal de Minas Gerais
